# Caffeine-induced increase in voluntary activation and strength of the quadriceps muscle during isometric, concentric and eccentric contractions

**DOI:** 10.1038/srep10209

**Published:** 2015-05-13

**Authors:** Martin Behrens, Anett Mau-Moeller, Matthias Weippert, Josefin Fuhrmann, Katharina Wegner, Ralf Skripitz, Rainer Bader, Sven Bruhn

**Affiliations:** 1 Department of Exercise Science, University of Rostock, Ulmenstrasse 69, 18057 Rostock, Germany; 2 Department of Orthopaedics, University Medicine Rostock, Doberaner Strasse 142, 18057 Rostock, Germany; 3 Institute of Exercise Physiology and Public Health, Trotzenburger Weg 15, 18057 Rostock, Germany

## Abstract

This study investigated effects of caffeine ingestion (8 mg/kg) on maximum voluntary torque (MVT) and voluntary activation of the quadriceps during isometric, concentric and eccentric contractions. Fourteen subjects ingested caffeine and placebo in a randomized, controlled, counterbalanced, double-blind crossover design. Neuromuscular tests were performed before and 1 h after oral caffeine and placebo intake. MVTs were measured and the interpolated twitch technique was applied during isometric, concentric and eccentric contractions to assess voluntary activation. Furthermore, normalized root mean square of the EMG signal was calculated and evoked spinal reflex responses (H-reflex evoked at rest and during weak isometric voluntary contraction) as well as twitch torques were analyzed. Caffeine increased MVT by 26.4 N m (95%CI: 9.3-43.5 N m, *P* = 0.004), 22.5 N m (95%CI: 3.1-42.0 N m, *P* = 0.025) and 22.5 N m (95%CI: 2.2-42.7 N m, *P* = 0.032) for isometric, concentric and eccentric contractions. Strength enhancements were associated with increases in voluntary activation. Explosive voluntary strength and voluntary activation at the onset of contraction were significantly increased following caffeine ingestion. Changes in spinal reflex responses and at the muscle level were not observed. Data suggest that caffeine ingestion induced an acute increase in voluntary activation that was responsible for the increased strength regardless of the contraction mode.

Caffeine is usually consumed in the form of tea, coffee, chocolate products as well as carbonated beverages and is one of the most popular drugs^1^. It has been shown that caffeine has an ergogenic effect on human performance. There is evidence that caffeine increases endurance and short-term, high-intensity exercise performance^2^,^3^. Early laboratory studies suggested an increased fat metabolism as a cause for better endurance performance after caffeine ingestion. However, later studies did not find any effect of caffeine on energy metabolism of the exercising muscle^4^. Thus, the ergogenic effect of caffeine on endurance exercise performance has been attributed to modulatory effects on the central nervous system. It has been argued, that caffeine reduces discomfort or pain during exercise and enables the subjects to exercise at higher intensities and/or for a longer time^5^. The caffeine-induced reduction in sensitivity to painful stimuli might be due to caffeine’s effect on peripheral and/or central adenosine A_1_ and/or A_2a_ receptors which are part of the nociceptive system. It is not unlikely, that caffeine also indirectly affects the nociceptive system by modulating muscle sensory processes and/or cardiovascular processes^6^.

The effect of caffeine on maximal voluntary contraction (MVC) strength is less clear. Several studies revealed an effect on MVC strength^7^,^8^ whereas others did not^9^,^10^,^11^. One explanation for the differing results could be that the ergogenic effect of caffeine seems to be muscle specific. Studies that have tested percent muscle activation and MVC strength of the knee extensors under isometric conditions have shown that caffeine is capable of increasing voluntary activation and strength^7^,^12^,^13^. However, studies that have analyzed the effect of caffeine on muscle activation and isometric MVC strength of the plantar flexors have not observed changes in these parameters^11^,^14^. A second explanation for the conflicting results could be that caffeine’s ergogenic effect might depend on the type of contraction, i.e. static vs. dynamic contractions. This assumption is based on the results of studies that have found a caffeine-induced enhancement of strength of the knee extensors under isometric conditions^7^,^12^,^13^, whereas no effect of caffeine on MVC strength was evident for isokinetic contractions^9^,^15^. Voluntary activation of muscles by the central nervous system generally depends on the excitability of cortical neurons and spinal α-motoneurons. However, the contribution of cortical and spinal centers to the neural drive differs depending on the type of contraction^16^. It has been shown that motor evoked potentials and H-reflexes evoked during eccentric voluntary contractions are smaller than those obtained during isometric and concentric voluntary contractions^17^,^18^,^19^. The published data on caffeine’s effect on cortical and spinal excitability, assessed during isometric voluntary contractions, indicate a central modulation rather than changes in the excitability of spinal α-motoneurons^7^,^11^. Thus, depending on the contraction mode and the associated contributions of central and spinal centers to the neural drive, caffeine should have distinct ergogenic effects on MVC strength. Previous research on voluntary muscle activation after caffeine ingestion using the interpolated twitch technique has been performed under isometric testing conditions only^12^,^7^. Consequently, nothing is known about changes in MVC strength of the quadriceps muscle and the underlying neural mechanisms during concentric and eccentric voluntary contractions after caffeine administration.

Therefore, we investigated the effects of caffeine ingestion on neuromuscular function of the quadriceps muscle during isometric, concentric and eccentric MVCs for the first time. In particular, the neural drive to the knee extensors during static and dynamic MVCs was measured by using the interpolated twitch technique and the root mean square of the EMG signal normalized to the maximal M-wave (M_max_). Furthermore, the putative caffeine-induced modulation of the excitability of spinal α-motoneurons via the Ia afferents was tested by eliciting H-reflexes in the vastus medialis (VM) at rest and during weak isometric voluntary contractions. Changes at the muscle level were assessed by analyzing the twitch torque signal induced by transcutaneous supramaximal electrical stimulation of the femoral nerve.

We hypothesized a caffeine-related increase in quadriceps MVC strength during isometric, concentric and eccentric contractions and an association between these changes and muscle activation as well as spinal excitability assessed by the H-reflex technique. Furthermore, we expected effects of caffeine on explosive voluntary strength and muscle activation at the onset of contraction. In addition, it was thought that contractile function of the knee extensors would not change.

## Results

Findings at baseline are given in Table 1. The H-reflexes evoked at rest and during 10% of isometric MVC strength were present in seven and 13 subjects, respectively. Due to subjective uncomfortable sensations during the eccentric contractions, four subjects declined to perform this contraction mode. Therefore, data of ten subjects are presented for the eccentric protocol.

**Figure 1:**
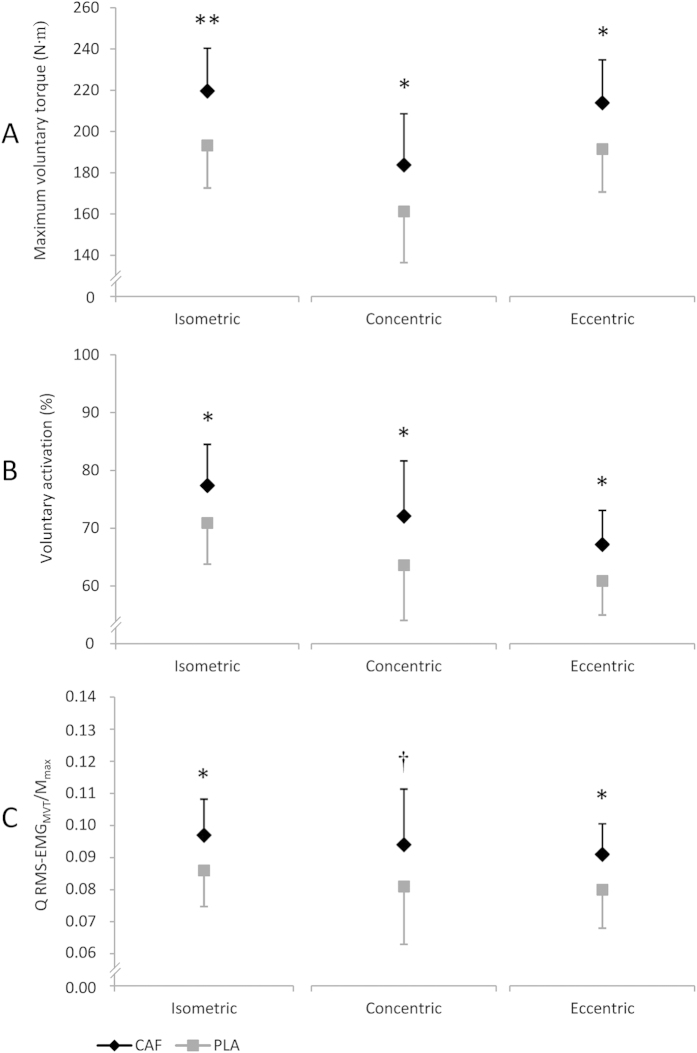
Effect of caffeine (CAF) on (**A**) maximum voluntary torque (iMVT), (**B**) voluntary activation and (**C**) normalized muscle activity of the quadriceps at MVT (Q RMS-EMG_MVT_/M_max_) during isometric, concentric and eccentric MVCs. ∗ denotes a significant difference between trials (ANCOVA with baseline-adjustment, ∗ *P*** ≤ **0.05; ∗∗ *P*** ≤ **0.01) and **†** denotes a statistical tendency towards a significant difference between trials (ANCOVA with baseline-adjustment, *P*** ≤ **0.09). PLA: placebo trial

After caffeine ingestion, isometric, concentric and eccentric maximum voluntary torques (MVTs) were significantly increased by 26.4 N m (9.3 to 43.5 N m, *P* = 0.004, η_p_^2^ = 0.289, ƒ = 0.638), 22.5 N m (3.1 to 42.0 N m, *P* = 0.025, η_p_^2^ = 0.186, ƒ = 0.478) and 22.5 N m (2.2 to 42.7 N m, *P* = 0.032, η_p_^2^ = 0.257, ƒ = 0.588), respectively, compared to placebo (Fig. 1A). The strength enhancements were associated with significant group-differences in voluntary activation during isometric, concentric and eccentric MVCs of 6.5% (0.9 to 12.1%, *P* = 0.024, η_p_^2^ = 0.188, ƒ = 0.481), 8.5% (1.1 to 16.0%, *P* = 0.027, η_p_^2^ = 0.181, ƒ = 0.470) and 6.3% (0.6 to 12.0%, *P* = 0.034, η_p_^2^ = 0.252, ƒ = 0.580), respectively (Fig. 1B). Normalized activity of the quadriceps muscle during isometric and eccentric MVCs (Q RMS-EMG_MVT_/M_max_) was significantly higher for the caffeine trial compared with placebo [0.011 (0.001 to 0.021, *P* = 0.039, η_p_^2^ = 0.159, ƒ = 0.435) and 0.011 (0.000 to 0.022, *P* = 0.050, η_p_^2^ = 0.220, ƒ = 0.531)]. Q RMS-EMG_MVT_/M_max_ during concentric MVCs showed a statistical tendency towards a significant difference between trials [0.013 (−0.002 to 0.028, *P* = 0.089, η_p_^2^ = 0.112, ƒ = 0.355)] (Fig. 1C).

**Figure 2:**
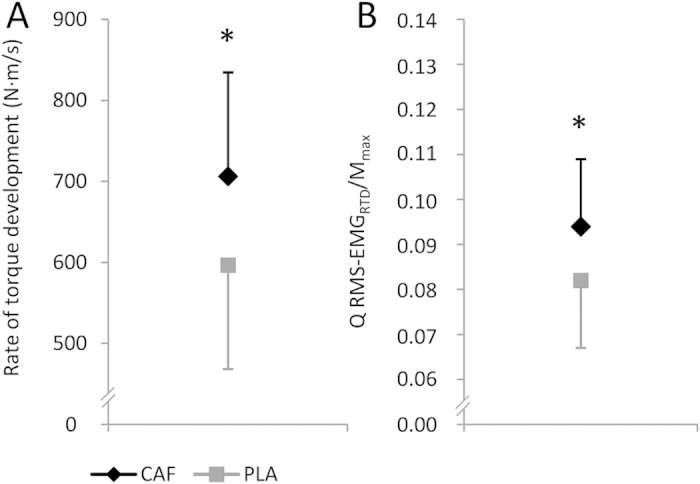
Effect of caffeine (CAF) on (**A**) rate of torque development (RTD) and (**B**) normalized muscle activity (Q RMS-EMG_RTD_/M_max_). ∗ denotes a significant difference between trials (ANCOVA with baseline-adjustment, ∗ *P*** ≤ **0.05). PLA: placebo trial

The slope of the torque-time curve in the time interval 0-200 ms during isometric MVCs was 109.6 N m s^−1^ (9.5 to 209.7 N m s^−1^, *P* = 0.033, η_p_^2^ = 0.169, ƒ = 0.451) steeper for the caffeine trial compared with placebo (Fig. 2A). Furthermore, muscle activation (RMS-EMG_RTD_/M_max_) in the initial phase of quadriceps contraction, i.e. 0-200 ms, was 0.011 (0.000 to 0.022, *P* = 0.048, η_p_^2^ = 0.148, ƒ = 0.417) higher at post-test for the caffeine trial compared with the placebo trial (Fig. 2B).

Resting twitch torques and evoked potentials were not different between trials (Table 2).

## Discussion

The present study analyzed the neuromuscular function of the knee extensors following caffeine and placebo ingestion. Data indicate that caffeine increased isometric, concentric and eccentric MVC strength due to an increased voluntary activation of the quadriceps muscle. In addition, the slope of the moment-time curve in the time interval 0-200 ms was steeper in the caffeine trial compared with the placebo trial. The increased explosive voluntary strength during the caffeine trial was accompanied by enhanced neural drive to the knee extensors, as evidenced by increased normalized muscle activity during torque development (RMS-EMG_RTD_/M_max_). Caffeine had no effect on the H-reflex elicited at rest and during weak isometric voluntary contractions as well as on peak twitch torques of the quadriceps.

To the best of our knowledge, this is the first study analyzing the effects of caffeine ingestion on MVC strength and voluntary activation of the quadriceps muscle during isometric, concentric and eccentric contractions. Isometric MVC strength was significantly enhanced in the caffeine trial compared to the placebo trial. This result is in accordance with the findings of previously published studies that reported an increased isometric MVC strength of the knee extensors after caffeine intake^7^,^12^,^13^. Concentric and eccentric MVC strength was enhanced as well, indicating that caffeine increased MVC strength regardless of the contraction mode. Our data on voluntary activation demonstrate that caffeine increased isometric, concentric and eccentric MVT of the knee extensors due to an augmented neural drive to the agonistic muscles. The voluntary activation during the different types of contraction seems to be relatively low, but is comparable to those observed during isometric MVCs of the knee extensors at a similar knee angle^20^. Normalized muscle activity was significantly higher or tended to be a higher for the caffeine trial and therefore supports the findings for voluntary activation data. These data and the unchanged peak twitch torque of the quadriceps muscle indicate that the acute adjustments were mainly of neural origin. Unfortunately, we were not able to differentially measure changes in the excitability of cortical and/or spinal neurons by using the interpolated twitch technique. However, our data on the H-reflexes evoked at rest and during weak isometric voluntary contractions indicate that the acute modulations occurred at the supraspinal level. Similar results, i.e. an unchanged H-reflex of the soleus muscle at rest and during MVC following caffeine administration, were observed previously^12^,^11^. Furthermore, it has been shown that the motor evoked potentials and the cortically evoked twitches of vastus lateralis during weak isometric voluntary contractions were increased following caffeine ingestion^7^ indicating a supraspinal site of the excitatory action of caffeine. The potential mechanisms include caffeine’s role as an adenosine receptor antagonist that reverses the tonic inhibitory influence of adenosine in the central nervous system like the adenosine-induced decrease in excitatory neurotransmitter release and firing rates of central neurons^21^. A study by Phillis *et al.*^22^ points in the same direction. The authors have revealed that caffeine enhances the firing rate of cerebral cortical neurons and lowers the threshold for neural activation. Furthermore, it has been shown that central adenosine A_2a_ receptor agonists depress the firing of cerebral cortical neurons and lead to hypoactivity, depression of locomotor activity and impairment of coordination^23^.

With regard to the effect of caffeine on explosive voluntary strength, data indicate that caffeine increased rate of torque development (RTD) of the knee extensors in the time interval 0–200 ms in physically active men and women due to increased neural drive to the agonistic muscles. This result is in accordance with the outcome of a recently published study by our group that has analyzed the effect of caffeine on neuromuscular function of the plantar flexors^11^. We have shown that the slope of the moment-time curve at the onset of an isometric MVC of the triceps surae was significantly steeper in the caffeine trial compared with the placebo trial. This change was accompanied by enhanced neural drive to the muscles. Because H-reflexes of the soleus muscle at rest and during MVC were not different between the caffeine and placebo trial, we hypothesized that caffeine might have altered cortico-spinal excitability exclusively at the onset of contraction which in turn could have enhanced normalized muscle activity of the triceps surae during torque development. This assumption corresponds with the results of other studies proposing a supraspinal site of the excitatory action of caffeine^12^,^7^,^22^.

In summary, this is the first study analyzing the effects of caffeine ingestion on MVC strength and voluntary activation of the quadriceps muscle during isometric, concentric and eccentric contractions. We have clearly shown that voluntary activation and, in turn, MVC strength were enhanced following caffeine ingestion regardless of the contraction mode. Furthermore, muscle activation during torque development and explosive voluntary strength during isometric contractions were increased as well. Data indicate that acute neural adaptations at the supraspinal and/or cortical level rather than changes in spinal excitability were responsible for the strength gains. It can be concluded that caffeine has an ergogenic effect on voluntary activation and strength of the knee extensors.

The results of the present study refer to the ergogenic effect of anhydrous caffeine. Therefore, the impact of caffeine on MVC strength and explosive voluntary strength of the quadriceps cannot be generalized to the effect of energy drinks or other caffeine-containing products.

## Methods

### Subjects

Fourteen healthy, non-smoking, physically active students (eleven males, three females, age: 25 ± 3 years, height: 179 ± 8 cm, body mass: 75 ± 12 kg) with no history of neurological disorders or injuries volunteered in the study. Although it is known that caffeine clearance can be decelerated by oral contraceptives^24^ and by hormonal fluctuations during the luteal phase of the menstrual cycle^25^ females were included in the study in order to test the effect of caffeine on neuromuscular function of the quadriceps in a mixed sample as done previously^15^,^13^,^11^. The participants were recreationally active (6 ± 3 h per week). Subjects’ self-reported caffeine consumption, assessed by the Caffeine Consumption Questionnaire, was 240 ± 172 mg per week. Persons with known medication, nutritional supplements or ergogenic aids were excluded from the experiment. Furthermore, the applicants were instructed to ensure a similar food intake on the days of the measurements. The sample size was similar to those in previous studies^14^,^26^,^11^. The study was a randomized, controlled, double-blind crossover trial. The treatment order (caffeine or placebo) was randomly assigned to participants. Randomization was performed by a computer-generated table of random numbers. Neither the investigators nor participants were aware of the treatment order. The treatment order was counterbalanced. Before testing, subjects were instructed to refrain from consuming alcohol and caffeine in the 72 h preceding the experiments and not to perform any strenuous exercise in the 72 h previous to the measurements. All persons were informed of the utilized procedures and signed informed consent prior to investigation. The study was conducted according to the declaration of Helsinki and was approved by the university’s ethics committee.

### Experimental procedure

**Figure 3:**
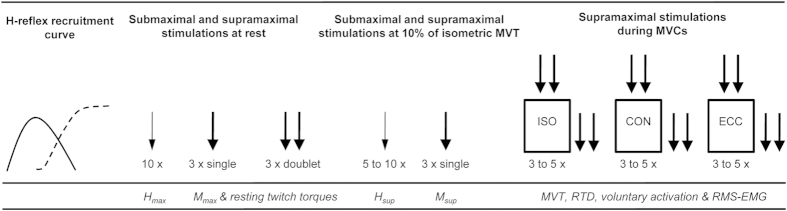
An overview of the procedures carried out during neuromuscular testing of the knee extensors and the extracted parameters. The thin arrow indicates electrical stimulation at submaximal intensity and the thick arrow indicates electrical stimulation at supramaximal intensity. H_max_: maximal H-reflex at rest, M_max_: maximal M-wave at rest, H_sup_: maximal H-reflex at 10% of MVC strength during an isometric voluntary contraction, M_sup_: maximal M-wave at 10% of MVC strength during an isometric voluntary contraction, MVT: maximum voluntary torque, RTD: rate of torque development, RMS-EMG: root mean square of the EMG signal, ISO: isometric, CON: concentric, ECC: eccentric.

All subjects took part in three experimental sessions. In the first session, subjects were familiarized with peripheral electrical nerve stimulation and different strength tests consisting of isometric, concentric and eccentric MVCs. The second and third experimental sessions took place one week apart at the same time of the day. Consequently, seven days constituted the washout period in order to avoid carry-over effects. In these two sessions, different neuromuscular tests were performed followed by oral administration of a drug capsule. After a time period of 1 h the measurements were repeated to analyze the effect of caffeine or placebo on neuromuscular function of the quadriceps muscle. It has been shown that a time period of 1 h is sufficient for caffeine to reach peak plasma concentrations^27^. The neuromuscular tests consisted of submaximal and supramaximal electrical stimulations of the femoral nerve at rest and during isometric, concentric and eccentric MVCs (Fig. 3). The contraction sequences were randomized. No warm-up was performed before the neuromuscular tests in order to avoid H-reflex and M-wave potentiation^28^. The measurements were made on the quadriceps muscle of the right leg. Throughout the testing sessions, the subjects were comfortably seated in a standardized position on a CYBEX NORM dynamometer (Computer Sports Medicine^®^, Inc., Stoughton, MA, USA). Before neuromuscular testing, the subjects sat passively on the dynamometer for ~10 min in order to minimize potentiation effects from walking to the laboratory.

### Electrical stimulation

Transcutaneous electrical stimulation of the femoral nerve in the femoral triangle was used to generate the H-reflex and M-wave recruitment curve. Prior to attaching the stimulation electrodes, the skin was prepared by shaving and cleaning the relevant area. A hand-held stimulation probe was used to locate the optimum site of stimulation. Subsequently, the femoral nerve was stimulated using a cathode ball electrode which was fixed to the subject’s femoral triangle. The anode was a self-adhesive electrode (35 × 45 mm, Spes Medica, Genova, Italy) attached over the greater trochanter. The electrical stimuli were single (1 ms duration, 400 V) and paired rectangular pulses (1 ms duration, 10 ms apart, 400 V) delivered by a constant-current stimulator (Digitimer^®^ DS7A, Hertfordshire, UK). The inter stimulus intervals were provided by a LABVIEW^®^ based program (Stimuli, Pfitec, Endingen, Germany). The testing procedure included electrical stimulation (inter stimulus interval was randomized between 6 and 7 s) with increasing current intensity until identification of peak-to-peak maximal H-reflex (H_max_) and M_max_ of VM. H_max_ and M_max_ were elicited and recorded ten (H_max_) and three times (M_max_). In addition, maximal H-reflexes (H_sup_) and maximal M-waves (M_sup_) were evoked at 10% of MVC strength during an isometric voluntary contraction. These evoked potentials were elicited five to ten times and three times, respectively. M_max_ and M_sup_ responses were evoked with supramaximal stimulation intensity (140%). Resting twitch torques were evoked prior to the MVCs using supramaximal single and doublet stimuli.

Voluntary activation during isometric, concentric and eccentric MVCs was assessed by using the interpolated twitch technique^29^. All measurements were performed at 70° knee flexion (0° = full extension). For the isometric condition, the supramaximal electrical stimuli (doublet) were delivered to the femoral nerve 2 s after torque onset, during the plateau phase, and 2 s after MVC. Concentric and eccentric MVC testing was done at a velocity of 25°/s^30^,^31^,^32^. During dynamic contractions, the supramaximal doublet was triggered automatically and delivered at a knee angle of 70° (0° = full extension). Subjects had to relax their knee extensors immediately after every single contraction and the lever arm moved again through the same range of motion with the same velocity. During this passive trial, supramaximal electrical stimuli were applied at 70° knee flexion (0° = full extension) as well. The time between the active trial (concentric or eccentric MVC) and the electrical stimulation during the passive trial was 6 s^32^. The stimuli were triggered by a LABVIEW^®^ based program (Stimuli, Pfitec, Endingen, Germany).

### EMG and torque recordings

Surface EMG electrodes (EMG Ambu^®^ Blue Sensor N) were used to record muscle activity. The self-adhesive electrodes were firmly attached to the shaved, abraded and cleaned skin over VM, rectus femoris (RF) and vastus lateralis (VL) of the right leg. The resistance between electrodes was measured with a digital multimeter (MY-68, McVoice, Braunschweig, Germany) and was kept below 5 kΩ. The electrodes were applied with a center-to-center distance of 2 cm over the middle of the muscle bellies. The recording electrodes were in line with the presumed direction of the underlying muscle fibres and the reference electrode was attached to the patella of the ipsilateral leg. The locations of the electrodes were marked on the skin during the investigation period. Signals were amplified (2500 x), band-pass filtered (10-450 Hz) and digitized with a sampling frequency of 3 kHz through an analog-to-digital converter (NI PCI-6229, National Instruments, Austin, TX, USA). Both, the EMG and torque signals were sampled at 3 kHz and stored on a hard drive for later analysis with a custom built LABVIEW^®^ based program (Imago Record, Pfitec, Endingen, Germany).

Torque signals were measured using a CYBEX NORM dynamometer (Computer Sports Medicine^®^, Inc., Stoughton, MA, USA). The individual positioning for each subject was similar during the three experimental sessions. The participants were seated with a hip joint angle of 80°. The measurements during the isometric condition were performed at 70° knee flexion. In the concentric and eccentric MVC trials, testing was performed between 90° and 5° knee flexion at a velocity of 25°/s, while the electrical stimuli were delivered at 70° (0° = full extension). The subjects’ knee joints were aligned with the axis of the dynamometer. The lever arm of the dynamometer was attached to the anterior aspect of the shank 2-3 cm above the lateral malleolus. The shin cushion was removed to avoid artifacts in the torque signal. A shin guard ensured that participants could exert maximal forces without discomfort. Straps across the waist and chest prevented excessive movements^32^.

During isometric, concentric and eccentric MVC strength testing, the subjects were instructed to exert maximal voluntary knee extensions against the lever arm of the dynamometer. For each trial, subjects were thoroughly instructed to act as forcefully and as fast as possible. They were motivated by strong verbal encouragement and online visual feedback about the instantaneous dynamometer torque provided on a digital oscilloscope (HM1508, HAMEG Instruments, Mainhausen, Germany). A rest period of at least 1 min was allowed between the trials. The maximal attempts were recorded until the coefficient of variance of the best three trials was below 5%^33^.

### Drug administration

Caffeine (Coffeinum N, Mylan dura GmbH, Germany) and placebo (all-purpose flour) of 8 mg/kg were administered in a gelatine capsule immediately after the pre-test in a randomized, double-blind and counterbalanced fashion. The caffeine dosage was similar to those in previous studies^14^,^26^,^11^. Intervention assignment was ascertained using envelopes with consecutive numbering. The person who opened the envelopes and carried out the assignments was not involved in data recording and data analysis. The subjects and the investigator were not informed about drug administration until the analysis was complete.

### Data analysis

The torque signals were corrected for the effect of gravity. Resting twitch torques were averaged and analyzed regarding their peak twitch torque, i.e. the highest value of twitch torque signal. H_max_, M_max_, H_sup_ and M_sup_ amplitudes were measured peak-to-peak and averaged, respectively. Furthermore, the H_max_/M_max_-ratio and H_sup_/M_sup_-ratio were calculated which can be considered as a global index of modulations at the spinal level due to alterations in α-motoneuron excitability and/or presynaptic inhibition of primary muscle spindle afferents^34^. The three best isometric, concentric and eccentric MVCs, respectively, were retained for analysis. On the basis of the torque-time curves of the MVC trials, MVT was determined, i.e. the highest torque value for the isometric contraction and the torque values immediately before the application of the electrical stimuli for the concentric and eccentric contractions. Explosive voluntary strength was determined on the basis of the torque-time curves of the isometric MVCs by analyzing the average RTD in the time interval of 0-200 ms relative to the onset of contraction.

Muscle activation during MVC was analyzed by calculating the root mean square of the amplitude of the EMG signal over a time interval of 200 ms at MVT (RMS-EMG_MVT_), i.e. 200 ms around the MVT for the isometric contraction and 200 ms prior to the electrical stimuli for the concentric and eccentric contractions. Muscle activation during the early phase of the isometric contractions was analyzed by calculating RMS-EMG_RTD_ in the time interval 0-200 ms relative to the onset of the EMG signals. In order to reduce errors due to electrode relocation and between-session variability in skin impedance, subcutaneous fat and fascia muscle activity was normalized, i.e. RMS-EMG of VM, RF and VL were normalized to the corresponding M_max_ values (RMS-EMG/M_max_). Furthermore, RMS-EMG/M_max_ was averaged across VM, RF and VL to calculate quadriceps activation at MVT (Q RMS-EMG_MVT_/M_max_) and during torque development (Q RMS-EMG_RTD_/M_max_).

The calculation of voluntary activation for the isometric contraction was done with the formula %VA = (1—superimposed twitch (T_b_/MVT) control twitch^−1^) 100^35^,^36^,^37^,^38^. T_b_ is the torque level immediately before the superimposed twitch. This formula counteracts the problem that, in some cases, the torque measured at the instant the superimposed doublet is delivered does not represent the maximal torque level. For the concentric and eccentric contractions, voluntary activation was calculated with the standard formula %VA = (1—superimposed twitch/control twitch) 100^29^.

### Statistical analysis

Data were checked for normal distribution using the Shapiro-Wilk test. The statistical approach comprised an analysis of covariance (ANCOVA) with baseline measurement entered as covariate^39^. The level of significance was established at *P* ≤ 0.05. SPSS 20.0 (SPSS Inc., Chicago, IL, USA) was used for statistical analysis. Data obtained at baseline are presented as mean values ± standard deviations and those obtained at the post-test are given as baseline-adjusted means ± baseline-adjusted standard deviations. If appropriate, data are presented as difference between means (95% confidence interval).

Effect size (ƒ) was calculated with the statistical software package G∗Power (version 3.1.4.)^40^. ƒ is a measure of the effectiveness of the intervention and it helps to determine whether a statistically significant difference is a difference of practical importance (ƒ = 0.10 small effect, ƒ = 0.25 medium effect, ƒ = 0.40 large effect)^41^.

## Author Contributions

M.B., A.M., M.W., J.F. and S.B. wrote the main manuscript. M.B., M.W., J.F., K.W., R.S. and R.B. participated in the experimental design and preparation of tables 1, 2 and figures 1, 2, 3. All authors reviewed the manuscript.

## Additional Information

**How to cite this article**: Behrens, M. *et al.* Caffeine-induced increase in voluntary activation and strength of the quadriceps muscle during isometric, concentric and eccentric contractions. *Sci. Rep.* **5**, 10209; doi: 10.1038/srep10209 (2015).

## Figures and Tables

**Table 1:** Peak twitch torques, evoked potentials, maximum voluntary torque (MVT), voluntary activation, normalized muscle activity during MVT (RMS-EMG_MVT_/M_max_), rate of torque development (RTD) and normalized muscle activity during RTD (RMS-EMG_RTD_/M_max_) in the time interval 0–200 ms for the caffeine (CAF) and placebo trial (PLA) obtained at baseline.

**Parameter**	**Baseline**		
	**CAF**	**PLA**	**Diff.**
***Peak twitch torques (N·m)***			
* Supramaximal single*	16.8 ± 10.4	17.7 ± 10.2	−0.9
* Supramaximal doublet*	38.6 ± 18.7	39.6 ± 18.1	−1.0
***Evoked potentials***			
* H*_*max*_ *vastus medialis (mV)*	1.72 ± 1.10	2.32 ± 1.96	−0.60
* M*_*max*_ *vastus medialis (mV)*	7.84 ± 3.87	7.86 ± 3.11	−0.02
* H*_*max*_*/M*_*max*_ *vastus medialis*	0.21 ± 0.12	0.24 ± 0.15	−0.03
* H*_*sup*_ *vastus medialis (mV)*	2.29 ± 2.13	1.94 ± 1.57	0.35
* M*_*sup*_ *vastus medialis (mV)*	7.46 ± 3.68	6.78 ± 2.90	0.68
* H*_*sup*_*/M*_*sup*_ *vastus medialis*	0.26 ± 0.14	0.26 ± 0.13	0.00
* M*_*max*_ *rectus femoris (mV)*	3.21 ± 1.53	3.09 ± 1.12	0.12
* M*_*max*_ *vastus lateralis (mV)*	6.20 ± 4.58	6.69 ± 4.64	−0.49
***Maximum voluntary torque (N·m)***			
* Isometric*	198.7 ± 37.4	211.3 ± 46.0	−12.6
* Concentric*	165.2 ± 42.0	179.6 ± 43.7	−14.4
* Eccentric*	203.5 ± 35.2	203.3 ± 40.4	0.2
***Voluntary activation (%)***			
* Isometric*	72.3 ± 10.2	73.8 ± 11.4	−1.5
* Concentric*	65.9 ± 12.7	68.5 ± 14.3	−2.6
* Eccentric*	62.2 ± 5.6	62.7 ± 11.3	−0.5
***RMS-EMG***_***MVT***_***/M***_***max***_***ISO***			
* Quadriceps*	0.086 ± 0.019	0.085 ± 0.018	0.001
* Vastus medialis*	0.062 ± 0.027	0.056 ± 0.017	0.006
* Rectus femoris*	0.109 ± 0.033	0.114 ± 0.037	−0.005
* Vastus lateralis*	0.088 ± 0.032	0.085 ± 0.027	0.003
***RMS-EMG***_***MVT***_***/M***_***max***_***CON***			
* Quadriceps*	0.081 ± 0.022	0.083 ± 0.020	−0.002
* Vastus medialis*	0.058 ± 0.025	0.054 ± 0.015	0.004
* Rectus femoris*	0.102 ± 0.036	0.114 ± 0.042	−0.012
* Vastus lateralis*	0.082 ± 0.032	0.082 ± 0.028	0.000
***RMS-EMG***_***MVT***_***/M***_***max***_***ECC***			
* Quadriceps*	0.081 ± 0.022	0.074 ± 0.010	0.007
* Vastus medialis*	0.056 ± 0.028	0.051 ± 0.015	0.005
* Rectus femoris*	0.100 ± 0.033	0.093 ± 0.021	0.007
* Vastus lateralis*	0.087 ± 0.028	0.078 ± 0.022	0.009
* Rate of torque development (N·m/s)*	623.5 ± 162.2	657.2 ± 188.3	−33.7
***RMS-EMG***_***RTD***_***/M***_***max***_			
* Quadriceps*	0.084 ± 0.021	0.084 ± 0.016	0.000
* Vastus medialis*	0.063 ± 0.024	0.059 ± 0.019	0.004
* Rectus femoris*	0.104 ± 0.036	0.107 ± 0.031	−0.003
* Vastus lateralis*	0.086 ± 0.032	0.085 ± 0.023	0.001

Diff.: difference between means, H_max_: maximal H-reflex at rest, M_max_: maximal M-wave at rest, H_sup_: H-reflex during 10% of isometric MVT, M_sup_: maximal M-wave during 10% of isometric MVT, ISO: isometric, CON: concentric, ECC: eccentric. Data are means ± standard deviations.

**Table 2:** Peak twitch torques, evoked potentials, normalized muscle activity during maximum voluntary torque (RMS-EMG_MVT_/M_max_) and normalized muscle activity during rate of torque development (RMS-EMG_RTD_/M_max_) in the time interval 0–200 ms for the caffeine (CAF) and placebo (PLA) trial obtained at post-tests.

**Parameter**	**Post**			
	**CAF**	**PLA**	**Diff. (95% CI)**	***P***
***Peak twitch torques (N·m)***				
* Supramaximal single*	17.3 ± 2.1	16.5 ± 2.1	0.8 (−0.8 to 2.4)	0.315
* Supramaximal doublet*	40.5 ± 2.6	39.9 ± 2.6	0.6 (−1.5 to 2.6)	0.608
***Evoked potentials (mV)***				
* H*_*max*_ *vastus medialis (mV)*	2.19 ± 1.32	1.77 ± 1.32	0.42 (−1.23 to 2.07)	0.583
* M*_*max*_ *vastus medialis (mV)*	7.85 ± 1.62	7.33 ± 1.62	0.52 (−0.74 to 1.78)	0.404
* H*_*max*_*/M*_*max*_ *vastus medialis*	0.21 ± 0.05	0.21 ± 0.05	0.00 (−0.06 to 0.06)	0.946
* H*_*sup*_ *vastus medialis (mV)*	1.67 ± 1.27	1.97 ± 1.27	−0.30 (−1.34 to 0.73)	0.547
* M*_*sup*_ *vastus medialis (mV)*	7.10 ± 2.01	7.14 ± 2.01	−0.04 (−1.62 to 1.54)	0.957
* H*_*sup*_*/M*_*sup*_ *vastus medialis*	0.20 ± 0.10	0.26 ± 0.10	−0.06 (−0.14 to 0.02)	0.163
* M*_*max*_ *rectus femoris (mV)*	3.02 ± 0.44	3.10 ± 0.44	−0.08 (−0.42 to 0.27)	0.654
* M*_*max*_ *vastus lateralis (mV)*	6.05 ± 1.02	6.47 ± 1.02	−0.42 (−1.21 to 0.38)	0.288
***RMS-EMG***_***MVT***_***/M***_***max***_***ISO***				
* Vastus medialis*	0.070 ± 0.019	0.062 ± 0.019	0.008 (−0.007 to 0.023)	0.304
* Rectus femoris*	0.122 ± 0.022	0.114 ± 0.022	0.008 (−0.011 to 0.026)	0.291
* Vastus lateralis*	0.098 ± 0.019	0.084 ± 0.019	0.014 (0.000 to 0.030)	**0.056**†
***RMS-EMG***_***MVT***_***/M***_***max***_***CON***				
* Vastus medialis*	0.066 ± 0.022	0.062 ± 0.022	0.004 (−0.012 to 0.021)	0.572
* Rectus femoris*	0.123 ± 0.030	0.105 ± 0.030	0.018 (−0.006 to 0.042)	0.130
* Vastus lateralis*	0.091 ± 0.019	0.078 ± 0.019	0.013 (−0.003 to 0.028)	0.107
***RMS-EMG***_***MVT***_***/M***_***max***_***ECC***				
* Vastus medialis*	0.073 ± 0.020	0.060 ± 0.020	0.013 (−0.007 to 0.033)	0.178
* Rectus femoris*	0.106 ± 0.017	0.103 ± 0.017	0.003 (−0.015 to 0.019)	0.797
* Vastus lateralis*	0.097 ± 0.018	0.076 ± 0.018	0.021 (0.004 to 0.038)	**0.021**∗
***RMS-EMG***_***RTD***_***/M***_***max***_				
* Vastus medialis*	0.066 ± 0.019	0.062 ± 0.019	0.04 (−0.009 to 0.018)	0.494
* Rectus femoris*	0.115 ± 0.022	0.104 ± 0.022	0.011 (−0.007 to 0.029)	0.224
* Vastus lateralis*	0.099 ± 0.019	0.081 ± 0.019	0.018 (0.003 to 0.033)	**0.023**∗

Diff. (95% CI): difference between means (95% confidence interval), H_max_: maximal H-reflex at rest, M_max_: maximal M-wave at rest, H_sup_: H-reflex during 10% of isometric MVT, M_sup_: maximal M-wave during 10% of isometric MVT, ISO: isometric, CON: concentric, ECC: eccentric. Data are baseline-adjusted means ± baseline-adjusted standard deviations.

^∗^denotes a significant difference between groups (ANCOVA with baseline-adjustment, P ≤ 0.05) and

^†^denotes a statistical tendency towards a significant difference between groups (ANCOVA with baseline-adjustment, P ≤ 0.06).
